# Identification of DRG-1 As a Melanoma-Associated Antigen Recognized by CD4^+^ Th1 Cells

**DOI:** 10.1371/journal.pone.0124094

**Published:** 2015-05-20

**Authors:** Yukiko Kiniwa, Jiang Li, Mingjun Wang, Chuang Sun, Jeffrey E. Lee, Rong-Fu Wang, Helen Y. Wang

**Affiliations:** 1 Center for Cell and Gene Therapy, Department of Pathology and Immunology, Baylor College of Medicine, Houston, Texas, United States of America; 2 Center for Inflammation and Epigenetics, Houston Methodist Research Institute, Houston, Texas, United States of America; 3 Department of Surgical Oncology, UT MD Anderson Cancer Center, Houston, Texas, United States of America; Ohio State University, UNITED STATES

## Abstract

Immunotherapy has emerged as a promising strategy for the treatment of metastatic melanoma. Clinical studies have demonstrated the feasibility of cancer immunotherapy using tumor antigens recognized by CD8^+^ T cells. However, the overall immune responses induced by these antigens are too weak and transient to induce tumor regression in the majority of patients who received immunization. A growing body of evidence suggests that CD4^+^ T helper (Th) cells play an important role in antitumor immunity. Therefore, the identification of MHC class II-restricted tumor antigens capable of stimulating CD4^+^ T cells may provide opportunities for developing effective cancer vaccines. To this end, we describe the identification of developmentally regulated GTP-binding protein 1 (DRG-1) as a melanoma-associated antigen recognized by HLA-DR11-restricted CD4^+^ Th1 cells. Epitope mapping analysis showed that the DRG1_248-268_ epitope of DRG-1 was required for T cell recognition. Reverse transcription-polymerase chain reaction revealed that DRG-1 was highly expressed in melanoma cell lines but not in normal tissues. DRG-1 knockdown by lentiviral-based shRNA suppressed melanoma cell proliferation and soft agar colony formation. Taken together, these data suggest that DRG-1 plays an important role in melanoma cell growth and transformation, indicating that DRG1 may represent a novel target for CD4^+^ T cell-mediated immunotherapy in melanoma.

## Introduction

Melanoma is the most aggressive form of skin cancer, with metastatic disease occurring in 10%–15% of patients at diagnosis [1], and is continuing to be a major health concern. The National Cancer Institute estimates that 76,100 Americans will be diagnosed with melanoma, and 9,710 will die from the disease in 2014. Metastatic melanoma has a dismal prognosis; the 5-year survival rates plummet from 98.2% for patients with localized disease to 61.7% and 15.2% for individuals with regional and distant metastases, respectively [2]. Current therapeutic options for metastatic melanoma are limited by low efficacy rates, toxic side effects, and drug resistance development [1,3,4]. Thus, new therapeutic strategies are urgently needed for the treatment of metastatic melanoma.

T cell-based immunotherapy has emerged as a promising strategy for the treatment of metastatic melanoma. Clinical trials using adoptive cell transfer with autologous tumor-reactive T cells have achieved encouraging results in patients with advanced melanoma [5–8], with evidence of durable, complete tumor responses. Since the success of cancer immunotherapy relies largely on the identification of suitable tumor-associated antigens (TAA) expressed by cancer cells [9], it has prompted the identification of melanoma-associated antigens recognized by T cells for the generation of cancer-specific T cells or vaccine development. However, most cancer vaccine trials have shown disappointing results [10]. One explanation may be the fact that most research has focused on the identification of tumor antigens recognized by MHC class I (MHC-I)-restricted CD8^+^ T cells, and many tumor antigens recognized by CD8^+^ T cells have proven to be poorly immunogenic. Increasing evidence has demonstrated that CD4^+^ T helper (Th) cells play a pivotal role in initiating and maintaining antitumor immune responses [11]. CD4^+^ T cells are required for the optimal expansion and effector function of CD8^+^ T cells [12–15]. Furthermore, CD4^+^ T cells have been shown to directly inhibit tumor growth and progression independent of their effects on CD8^+^ T cells [12,13,16–19]. These insights indicate that optimal vaccination may require the participation of both CD4^+^ and CD8^+^ T cells to generate a strong and long-lasting antitumor immunity. Therefore, the identification of MHC class II-restricted tumor antigens, which can stimulate CD4^+^ T cells, may provide opportunities for developing effective cancer vaccines.

Herein, we describe the identification and characterization of developmentally regulated GTP-binding protein 1 (DRG-1) as a melanoma-associated antigen recognized by HLA-DR11-restricted CD4^+^ Th1 cells. The DRG-1_248_ peptide was identified as the epitope required for CD4^+^ T cell recognition. DRG-1 was highly expressed in most melanoma cell lines, whereas its expression was low or absent in normal tissues. Gain-of-function and shRNA knockdown experiments revealed that DRG-1 promotes the proliferation and transformation of melanoma cells. Together, our findings indicate that DRG-1 may represent a novel target for melanoma immunotherapy. Thus, our study has important implications for the development of anticancer vaccines incorporating both MHC-I- and MHC-II-binding epitopes for melanoma immunotherapy.

## Materials and Methods

### Tumor cell lines, T cell lines/clones, and T cell expansion

To generate tumor-reactive T cell lines, CD4^+^ 155 tumor-infiltrating lymphocytes (TILs) were established from a melanoma patient. Melanoma tissues were obtained from patients who had signed informed consent. This protocol and study was approved by the Institutional Review Board (H9086) at MD Anderson Cancer Center and Baylor College of Medicine. Tissues were washed in RPMI 1640 medium, minced into small pieces, and digested with a triple enzyme mixture (1 mg/ml collagenase type IV, 0.1 mg/ml hyaluronidase, and 30 U/ml deoxyribonuclease in RPMI 1640 medium supplemented with 100 U/ml penicillin, 100 μg/ml streptomycin, 100 μg/ml gentamycin chloride, and 0.25 μg/ml fungizone) for 2 h at room temperature. After digestion, the cells were filtered with a 40-μm cell strainer and washed twice in RPMI 1640 medium. For the generation of tumor cell lines, cells were cultured in RPMI 1640 medium supplemented with 10% fetal calf serum (FCS), penicillin, and streptomycin. For the generation of T cells, cells were cultured in RPMI 1640 medium supplemented 10% human serum, 0.3 mg/ml L-glutamine, 55 μM 2-mercaptoethanol, and 1000 IU/ml interleukin-2 (IL-2). To generate tumor-reactive T cell lines, limiting dilution cloning was performed as previously described [20]. T cells (0.3 X 10^6^ cells per well) were seeded with irradiated peripheral blood mononuclear cells (PBMCs) isolated from fresh buffy coats from healthy donors obtained from a blood bank (Gulf Coast Regional Blood Center, Houston, TX) in RPMI 1640 medium supplemented with 10% human serum, 0.3 mg/ml L-glutamine, 55 μM 2-mercaptoethanol anti-CD3, and anti-CD28 antibodies (30 ng/ml). The next day, 50 IU/ml IL-2 was added. T cell clones were further expanded as previously described [21]. Melanoma cell lines and Epstein-Barr virus (EBV)-transformed B cell lines established in our Lab were maintained in RPMI 1640 with 10% FCS. 293IMDR11 cells were established by transfecting plasmid DNA encoding DRB1*1101 cDNA into 293 ECII cells [22].

### cDNA library construction and screening

Total RNA was extracted from 155mel cells using TRIzol reagent (Invitrogen). Poly(A) RNA was purified from total RNA using the polyATract system (Promega) and converted to cDNA using a cDNA construction kit (Invitrogen) with an oligo-dT primer. The cDNA inserts were then ligated to a pTSX vector containing an Ii fragment (aa 1–80), and the cDNA library was electroporated into DH10B cells. Plasmid DNA for the cDNA library was prepared from bacteria, each consisting of ~100 cDNA clones. DNA transfection was performed, as previously described [22]. Briefly, cDNA (0.2 μg) from each pool was mixed with Lipofectamine 2000 and added to 293IMDR11 cells (5 x 10^4^ cells/well) in 96-well plates and incubated for 24 h. The next day, CD4^+^ T cells (5 x 10^4^ cells/well) were added to 293IMDR11 cells. After 20 h, culture supernatant was collected, and granulocyte-macrophage colony-stimulating factor (GM-CSF) concentration was measured using a standard ELISA (Pierce).

### Peptide synthesis and T cell epitope mapping

To identify T cell epitopes, DRG-1 was truncated and tested for T cell recognition. DRG1 fragments A to F were generated by PCR using primers with adaptor sequences, 5’-CCGCTCGAGTCACTGGATCTTGGCACCTTTG-3’, 5’-CCGCTCGAGTCACGTAGAGTCACATCGCA-3’, 5’-TGCGCTAGCAGTGATGCTACGGCTGAT-3’, 5’-CCGCTCGAGTCAGTCCTTCTTAAAGCC-3’, 5’-CCGCTCGAGTCAGATATCCAATTACTCAAT-3’ and 5’-CGGAATTCCAAGGGAGGCATTAATCTCACA-3’. Only fragment G was generated by NheI and EcoRI digestion. Subsequently cDNA fragments were ligated into a pTSX vector containing an Ii fragment. T cell epitope candidates were predicted using the SYFPEITHI T cell epitope prediction tool. Antigenic peptides were synthesized by a solid-phase method using a peptide synthesizer (model AMS 422, Gilson). Peptides were purified by HPLC and had >98% purity. The mass of the peptides was confirmed by mass spectrometry analysis. Synthesized peptides were dissolved in DMSO to 10 mg/ml. 293DR11 cells were pulsed with antigenic peptides and co-cultured with TIL155-C1 cells overnight. T cell reactivity was assessed by interferon-γ (IFN-γ) release.

### Flow cytometric analysis of CD4 and CD8 expression

To analyze the tumor-reactive T cell type, T cells were washed in phosphate-buffered saline (PBS)/5% FCS, incubated with anti-CD4 or anti-CD8 antibodies at 4°C for 30 min, washed, and resuspended in PBS/5% FCS. Cells were analyzed using a FACScan flow cytometer (BD Biosciences) with CellQuest Pro software (BD Biosciences).

### Antibody blocking and cytokine release assays

To determine the MHC restriction of T cell recognition, T cell activity was measured in the absence or presence of various antibodies as previously described [22]. The antibodies, including L243 (anti-HLA-DR; HB55), IVA12 (anti-HLA-DR, DP, DQ; HB145), IVD12 (anti-HLA-DQ, DR; HB144), and W6/32 (HLA-A, B, C; HB95), were purified from American Type Culture Collection hybridoma supernatants. Tumor cells (2 x 10^4^) in 80 μl of T cell assay medium (RPMI 1640/2% human serum /20 IU IL-2) were incubated with 20 μl of an antibody (200 μg/ml) for 1 h. T cells (5 x 10^4^) in 100 μl of T cell assay medium were added and incubated overnight. Cytokines were measured in culture supernatants using standard ELISA kits (Pierce).

### Reverse transcription-polymerase chain reaction (RT-PCR) and northern blot analysis

RT-PCR was performed to evaluate the mRNA level of *DRG-1* (Forward primer: 5’-TGCGCTAGCAGCAGCACCTTAGCTAAGATC-3’, Reverse primer: 5’-CCGCTCGAGTCACGTAGAGTCACATCGGCA-3’) in melanoma cell lines and normal tissues. Total RNA was isolated from PBMCs, EBV-B cells, 293T cells, and melanoma cell lines using TRIzol reagent (Invitrogen). Total RNAs from various normal adult human tissues were purchased from Clontech. cDNA synthesis was performed using the Superscript II RT Kit (Invitrogen) according to the manufacturer’s instructions.

### Knockdown of *DRG-1* by short hairpin RNA (shRNA)

The target sequences for *DRG-1* shRNA oligos were 5´-GTATCTATGTGTTAAATAA-3’ (shRNA1) and 5´-GGTGATGCTCGAATTGAAT-3’ (shRNA2). The functional role of DRG-1 was assessed by transfecting control (5’-GCCCTTCATTGTAGATCTGA-3’) and *DRG-1*-specific shRNAs into 155mel cells. *DRG-1* and control shRNAs were cloned into the *pLL3*.*7* vector and transfected into 155mel cells. To demonstrate shRNA knockdown efficiency, the *DRG-1* gene was cloned into HA-tagged *pcDNA3*.*1* and transfected into 293T cells expressing *DRG-1*-specific or control shRNA. DRG-1 expression was analyzed by western blot.

### Cell proliferation

To assess the effect of DRG-1 on cell proliferation, tritiated [^3^H]thymidine uptake assays were performed on *DRG-1* shRNA-transfected 155mel cells as previously described [23]. Parental, empty-vector transfected, and control shRNA-transfected 155mel cells were used as controls.

### Soft agar colony formation assay

To determine the involvement of DRG-1 in malignant transformation, the soft agar colony formation assay was used. Cells (5 x 10^3^ cells/well) were mixed in 0.4% agarose gel and plated on top of a bottom layer of 0.8% agarose in RPMI 1640 medium supplemented with 10% FCS. After 10 days, cell colonies were stained with 0.5 mg/ml thiazole blue tetrazolin bromide (Sigma) and counted.

### Statistical analysis

Unless otherwise indicated, data are expressed as the mean ± standard deviation. Significant differences between groups were determined by Student’s t test, and a P value of less than 0.05 was considered significant.

## Results

### Establishment and characterization of tumor-specific CD4^+^ T cell lines/clones

**Fig 1:**
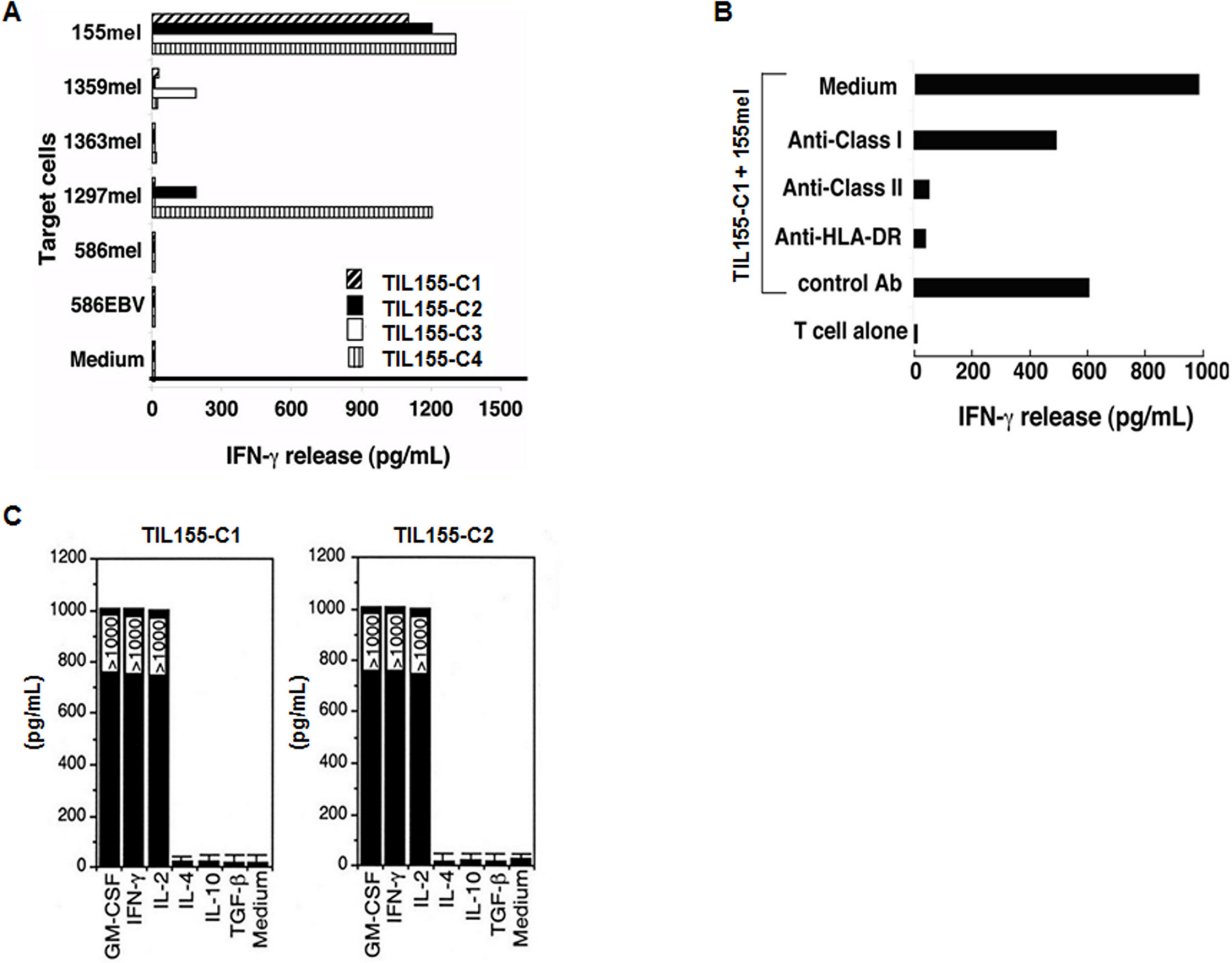
Specific recognition of autologous melanoma cells by CD4^+^ TIL155 clones. (A) Specific antitumor recognition of CD4^**+**^ TIL155-C1-4. The reactivity of four CD4^**+**^ TIL155 T cell clones to autologous 155mel cells, allogeneic melanoma cell lines, and the 586EBV-B cell line was evaluated based on IFN-γ release. (B) HLA restriction of T cell recognition. CD4^**+**^ TIL155-C1 cells were co-cultured with autologous 155mel cells in the presence or absence of various anti-MHC antibodies. IFN-γ release was determined after an 18-h incubation. (C) Cytokine profiles, measured by ELISA, of TIL155-C1 and TIL155-C2 cell culture supernatants after 155mel tumor cell stimulation.

TIL155 was established from a melanoma patient (HLA-DR*0101, *1101). Flow cytometric analysis demonstrated that TIL155 T cells were CD4^+^ (data not shown). To determine whether CD4^+^ TIL155 recognized specific tumor antigens, we generated 31 T cell clones by a limiting dilution method [21,24]. We selected four T cell clones/sublines for further expansion and subsequent studies. All four T cell clones were CD4^+^ T cells and recognized autologous 155mel tumor cells. TIL155-C2 and C4 also recognized allogeneic 1297mel tumor cells, whereas C3 recognized allogeneic 1359mel cells (Fig 1A). To determine the restriction elements of T cell recognition, TIL155 T cell activity was determined following pretreatment with various anti-HLA antibodies. The activity of TIL155-C1 T cells against autologous tumor cells was specifically blocked by anti-MHC class II and anti-HLA-DR monoclonal antibodies, but not by an anti-MHC class I antibody (Fig 1B). TIL155-C1 and TIL155-C2 cells had a cytokine signature characteristic of Th1 cells [25]. TIL155-C1 and TIL155-C2 secreted GM-CSF, IL-2, and IFN-γ but did not secrete IL-4, IL-10, or transforming growth factor (TGF)-β after exposure to 155mel tumor cells (Fig 1C). Together, these findings suggested that the TIL155 T cell clones were CD4^+^ Th1 cells, and their antigen specificity was either HLA-DR*0101- or HLA-DR*1101-restricted.

### Identification of DRG-1 as a tumor antigen recognized by HLA-DR11-restricted CD4^+^ T cells

**Fig 2:**
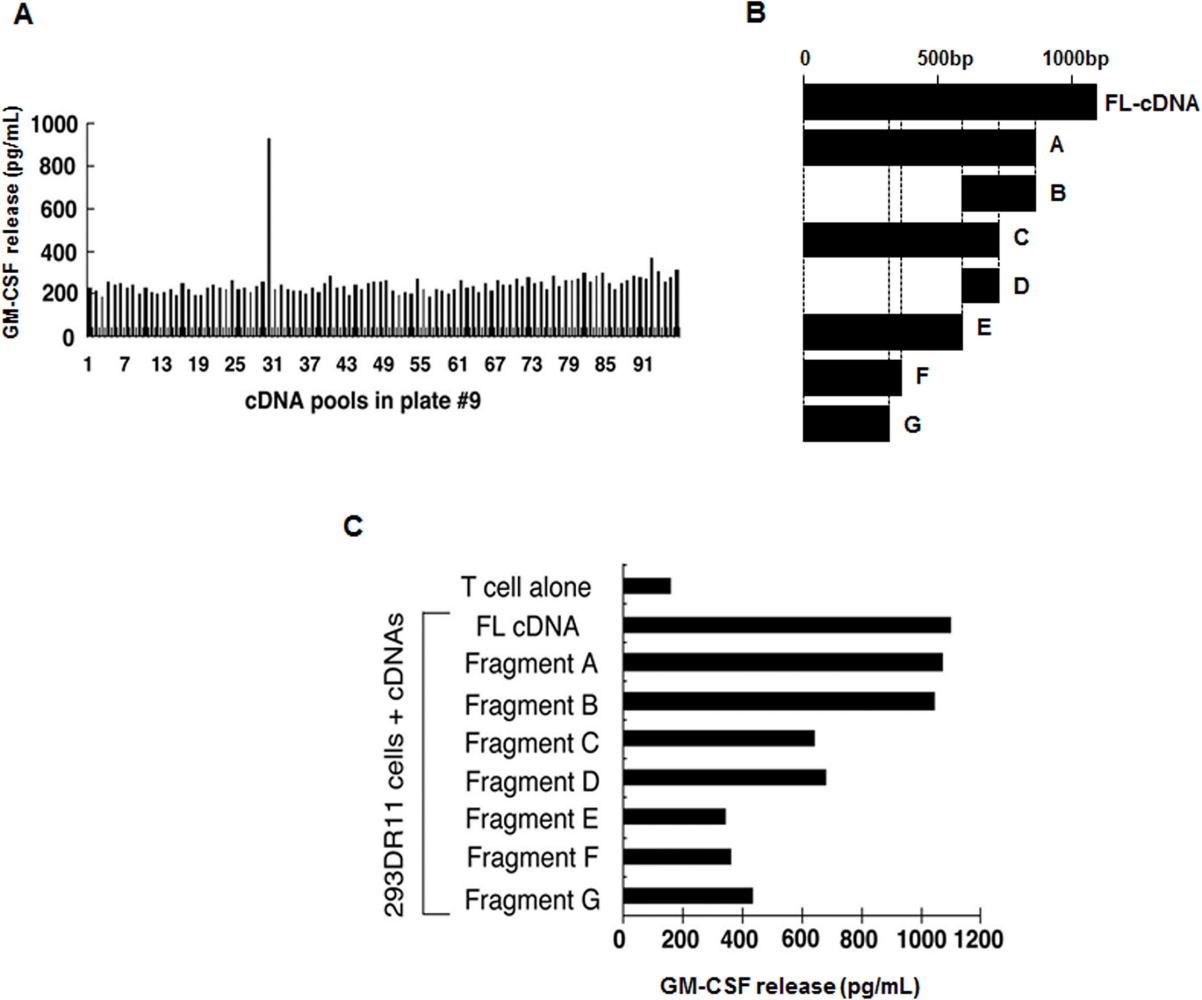
Ii-cDNA library screening using CD4^+^ TIL155-C1 cells. (A) Identification of a positive cDNA pool capable of stimulating CD4^**+**^ T cells. After screening 2 x 10^**5**^ Ii-cDNA fusion library clones generated from 155mel RNA, positive cDNA pools were identified based on GM-CSF release from CD4^**+**^ TIL155-C1 cells. (B) Schematic presentation of full-length (FL) *DRG-1* cDNA and its deletion constructs. Seven deletion constructs containing cDNA fragments of different lengths (A to G) were generated to identify HLA-DR11-restricted T cell epitopes from DRG-1. (C) T-cell activity of HLA-DR11-restricted T cell epitopes from DRG-1. The full-length (FL) *DRG-1* cDNA and seven deletion constructs (A to G) were transfected into 293IMDR11 cells and tested for T cell reactivity based on GM-CSF release.

To identify the genes encoding the tumor antigens recognized by CD4^+^ tumor-specific T cells, we used a genetic targeting expression system, which has been used to identify several MHC class II-restricted tumor antigens [20,22,26]. Because T cell reactivity against 155mel tumor cells was specifically blocked by an anti-HLA-DR monoclonal antibody and 155mel cells expressed HLA-DR*0101 and DR*1101, we determined TIL155-C1 T cell reactivity to 293IMDR1 cells transfected with an Ii-fused cDNA library constructed from 155mel RNA. After screening 1.7 x 10^5^ cDNA clones, no positive pool was identified, suggesting that the restriction element, HLA-DR1, was not recognized by TIL155-C1 T cells. Therefore, we established a new 293 cell line expressing HLA-DR11 (293IMDR11). After screening the same cDNA library (2 x 10^5^), one positive cDNA pool was identified, which contained approximately 100 independent cDNA clones (Fig 2A). The positive-pool DNA was subsequently transformed into *E*. *coli*, and individual colonies were picked for plasmid DNA preparation. After re-screening 200 individual plasmid DNAs, five single cDNA clones capable of stimulating T cell cytokine release were isolated. All five single cDNA clones contained the same size cDNA insert upon digestion with *NotI* and *BstXI* (data not shown). DNA sequencing analysis and database searches revealed that the cDNA clones were completely identical to the published sequence of the developmentally regulated GTP-binding protein-1 (DRG-1). These results indicated that TIL155-C1 CD4^+^ T cells recognize the non-mutated *DRG-1* gene product.

### Identification of the T cell epitope of DRG-1

**Fig 3:**
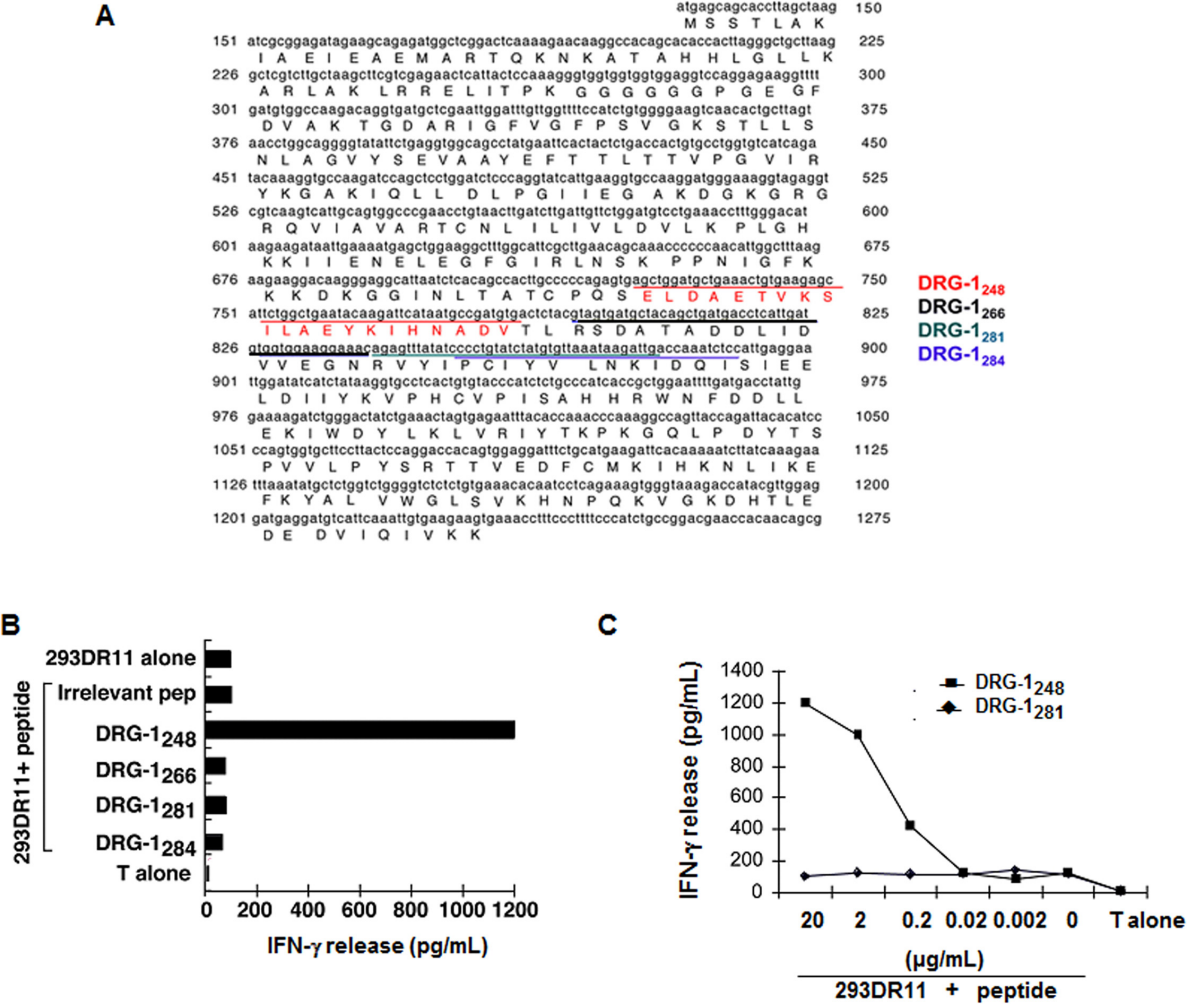
Identification and characterization of DRG-1 peptides capable of stimulating CD4^+^ T cells. (A) Four putative DRG-1 peptides recognized by TIL155-C1 cells. (B) Identification of the DRG-1 peptide required for T cell recognition. 293DR11 cells were pulsed with four DRG-1 and irrelevant peptides and tested for T cell recognition based on IFN-γ release from TIL155-C1 cells. (C) Determination of peptide concentrations required for T cell recognition. 293IMDR11 cells were incubated with the indicated concentrations of DRG-1_248_ and DRG-1_281_ peptides for 90 min and washed 3 times with T-cell assay medium. T cells were added to peptide-pulsed 293IMDR11 cells overnight. IFN-γ release from T cells was determined by ELISA.

To identify HLA-DR11-restricted T cell epitopes from DRG-1, we generated 7 constructs containing *DRG-1* cDNA fragments of different lengths (labeled A to G) (Fig 2B). The constructs were transfected into 293IMDR11 cells and tested for T-cell reactivity based on GM-CSF release. The cDNA constructs, A to D, were capable of stimulating GM-CSF secretion from TIL155-C1 T cells. In contrast, TIL155-C1 T cells exhibited little reactivity to constructs E to G as evidenced by the low GM-CSF levels (Fig 2C). These findings suggested that T cell epitopes were located between constructs D and E (i.e., cDNA nucleotide positions 575 to 770). Using a computer-assisted MHC class II epitope prediction program (SYFPEITHI), four peptides containing an HLA-DR11 binding motif were identified (Fig 3A) and tested for T cell reactivity. The DRG-1_248_ peptide strongly stimulated cytokine release from TIL155-C1 T cells, whereas DRG-1_266_, DRG-1_281_, and DRG-1_284_ failed to do so (Fig 3B). These results indicated that TIL155-C1 T cells recognize the DRG-1_248_ peptide in the context of HLA-DR11 molecules. Peptide titration experiments showed that DRG-1_248_ could be recognized by TIL155-C1 T cells at a concentration of 0.1 μM (Fig 3C). However, other TIL155 clones failed to recognize DRG-1 or DRG-1-derived peptides (data not shown), suggesting that they recognize additional antigens expressed on 155mel cells.

### *DRG-1* is highly expressed in melanoma cells

**Fig 4:**
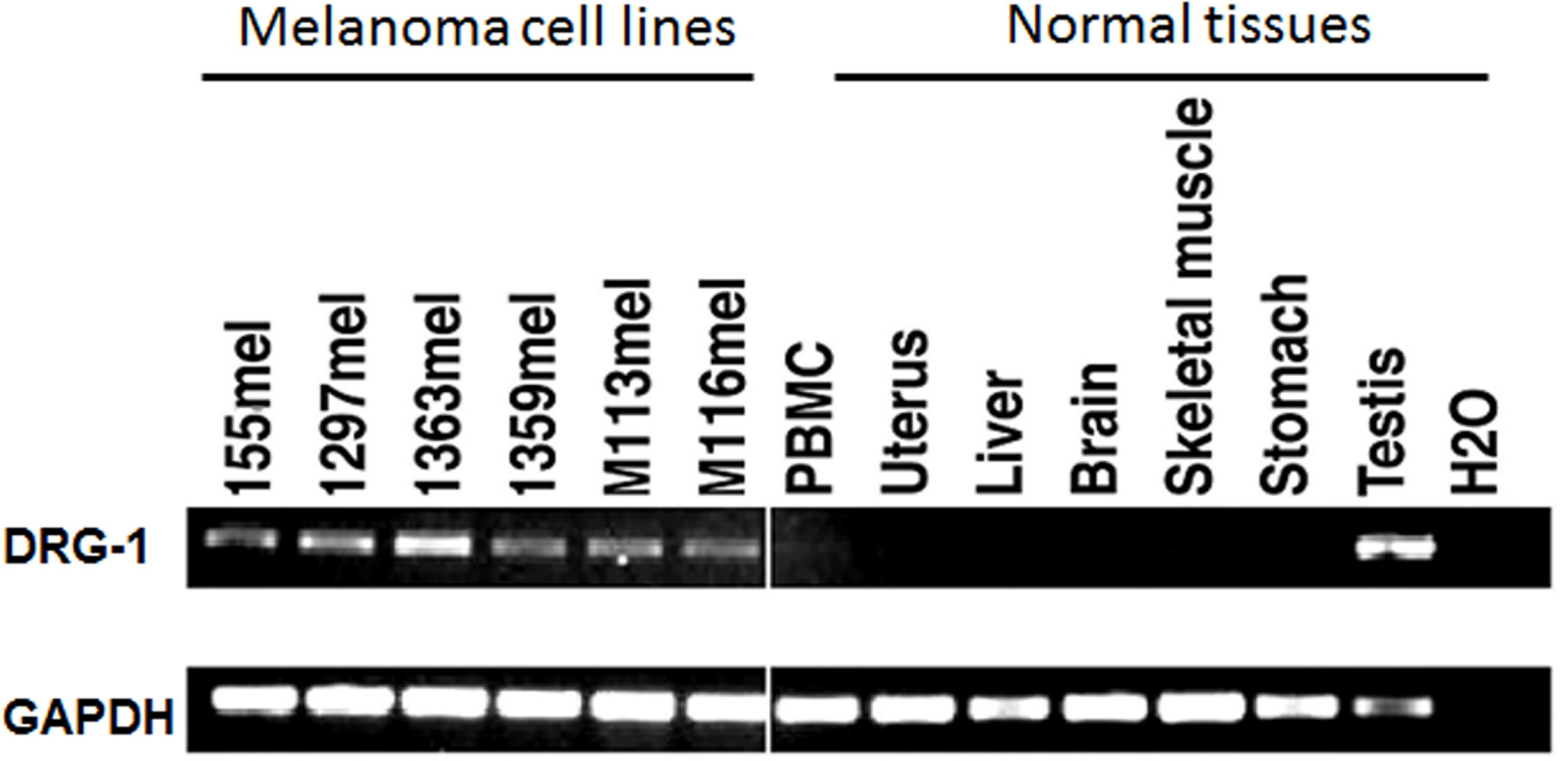
Expression pattern of *DRG-1* in melanoma cell lines and normal tissues. RT-PCR was performed to determine *DRG-1* expression in melanoma cell lines and normal tissues. GAPDH was used as a loading control.

The expression pattern of *DRG-1* was determined in melanoma cell lines and normal tissues using RT-PCR. *DRG-1* was highly expressed in most melanoma cell lines, whereas *DRG-1* expression was low or absent in normal tissues, with the exception of normal testis (Fig 4).

### *DRG-1* promotes proliferation and transformation of melanoma cells

**Fig 5:**
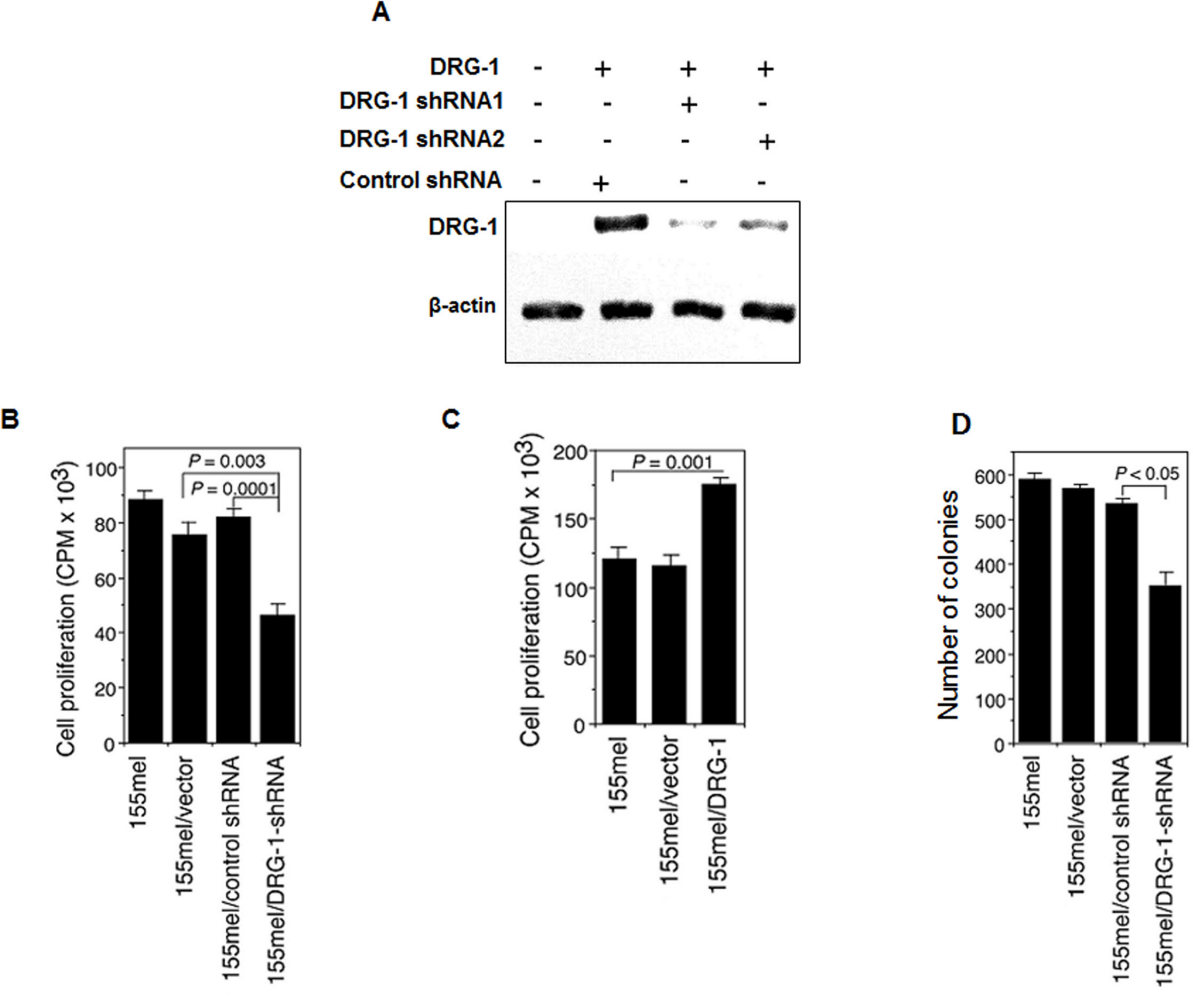
Knockdown of *DRG-1* reduces cell growth and transformation potential. (A) shRNA-mediated knockdown of *DRG-1*. *DRG-1*-specific lentiviral shRNAs were constructed and transfected into *DRG-1*-expressing 293T cells. Knockdown efficiency was determined by western blot analysis. β-actin was used as a loading control. (B) Proliferative ability of *DRG-1* knockdown melanoma cells. Cell proliferation was determined by [^**3**^H]thymidine incorporation in 155mel cells transfected with either control or *DRG-1*-specific shRNAs. Data are expressed as 10^**3**^ counts per minute (CPM). (C) Proliferative ability of *DRG-1*-overexpressing melanoma cells. Cell proliferation as assessed by [^**3**^H]thymidine uptake in 155mel cells transfected with either a retrovirus vector expressing *DRG-1* or empty vector. (D) Soft agar colony formation assay was used to determine the transformation ability of 155mel cells transfected with either control or *DRG-1*-specific shRNAs.

The elevated expression of DRG-1 in melanoma cells suggested the potential involvement of DRG-1 in the oncogenic process in melanoma. To test this possibility, a DRG-1 knockdown melanoma cell line was established using lentiviral-based shRNAs, and the proliferative and transformation abilities of this cell line were evaluated. Specific and efficient knockdown of DRG-1 was achieved in DRG-1-expressing 293T cells transfected with DRG-1-specific shRNAs (Fig 5A). Cell proliferation was significantly decreased in DRG-1 knockdown 155mel cells compared with empty vector-transfected and control shRNA-transfected cells (P = 0.003 and P = 0.0001, respectively; Fig 5B). In contrast, DRG-1 overexpression in 155mel cells significantly enhanced cell proliferation compared with control cells (P = 0.001; Fig 5C). DRG-1 knockdown significantly inhibited the transformation ability of melanoma cells as evidenced by the lower colony number compared with control cells (P < 0.05; Fig 5D). Taken together, these data suggested that DRG-1 plays an important role in melanoma development and progression.

## Discussion

Identification of genes encoding tumor-specific antigens recognized by T cells has led to the development of antigen-specific vaccines for cancer immunotherapy, which has emerged as a promising approach for cancer treatment [27–30]. Recent FDA approval of immunotherapy-based vaccines/drugs sipuleucel-T (Provenge) [31], ipilimumab (Yervoy) [32] and Keytruda [33] represent milestones in the field of cancer immunotherapy for advanced prostate cancer and metastatic melanoma, respectively [29]. It is widely accepted that successful cancer immunotherapy relies largely on the identification of suitable tumor-associated antigens [9]. To date, tumor antigen identification has primarily focused on antigens recognized by tumor-specific CD8^+^ T cells. Although a phase III clinical trial of gp100 peptide vaccine in patients with advanced melanoma has shown encouraging results [34], most tumor-specific CD8^+^ T cell responses induced by vaccination have not demonstrated effective tumor regression [10]. CD4^+^ Th cells have been shown to play a central role in antitumor immunity [11]. CD4^+^ T cells are critical for priming tumor-specific CD8^+^ T cells [13], generating and maintaining long-term CD8^+^ memory T cell responses, and controlling CD8^+^ T cell trafficking to tumor sites [13,35]. Furthermore, the combined administration of human CD4^+^ and CD8^+^ T cells has been shown to enhance the efficacy of antitumor responses compared with the administration of CD4^+^ or CD8^+^ T cells alone [36,37]. CD4^+^ T cells can also mediate tumor regression in the absence of CD8^+^ T cells, as shown by the adoptive transfer of tumor-specific CD4^+^ T cells [12,18,19]. Their role has been documented in many other tumor models [16], including CD4-knockout animals, which fail to control tumor outgrowth [13]. The mechanisms by which CD4^+^ T cells mediate tumor rejection are not clear. Several studies have suggested that the antitumor activities of CD4^+^ T cells are dependent on cytokines, such as IFN-γ and tumor necrosis factor-α [38,39]. Other studies have proposed that CD4^+^ T cells eliminate tumors through the activation and recruitment of effector cells, including macrophages and eosinophils [12,13]. Together, these studies highlight the importance of CD4^+^ T cells in achieving optimal antitumor immunity. Therefore, identification of tumor antigens recognized by CD4^+^ T cells may be beneficial to the development of successful vaccination strategies.

Our study identified DRG-1 as a melanoma-associated antigen capable of activating CD4^+^ Th1 cells. Although most tumor-associated antigens are not required for tumor growth, gain-of-function and shRNA-mediated knockdown experiments demonstrated the critical role of DRG-1 in the growth and proliferation of melanoma cells. Thus, DRG-1-specific Th1 cells may play a role in controlling tumor growth in melanoma patients. Tumor antigens that are essential for tumor cell survival and growth may better prevent the immunoselection of antigen-loss variants as a result of vaccination, thereby improving cancer immunotherapy efficacy [40,41]. Such immunogenic tumor antigens, eliciting minimal immune escape, may represent the most optimal vaccine candidates for cancer immunotherapy.

DRG-1 expression is increased in many types of human tumors including colon, breast, prostate, kidney, liver, and brain cancers [42]. In the present study, *DRG-1* was highly expressed in melanoma cell lines, whereas *DRG-1* expression was low or absent in normal tissues with the exception of normal testis. To our knowledge, our study is the first to report the expression of *DRG-1* in melanoma. We found that DRG-1 promoted the proliferation and anchorage-independent growth of melanoma cells. Anchorage-independent growth is associated with the tumorigenic and metastatic potential of tumor cells *in vivo*. Therefore, our findings strongly suggest the involvement of DRG-1 in melanoma development and progression. Consistent with our findings, *DRG-1* knockdown decreased non-small cell lung cancer (NSCLC) tumor growth *in vivo* [43]. DRG-1 has also been shown to be a predictor of poor prognosis in hepatocellular carcinoma, NSCLC, and cervical adenocarcinoma [43–45]. In contrast, DRG-1 has been shown to suppress the metastasis of colon and prostate cancers in the *in vivo* mouse models [46,47]. Furthermore, DRG-1 expression was reduced in clinical samples from breast cancer patients with metastasis to lymph nodes and bones [48]. DRG-1 overexpression has also been associated with favorable clinical outcome in patients with breast cancer, prostate cancer, esophageal squamous cell carcinoma, pancreatic cancer, and neuroblastoma [46,48–52]. These contradictory findings may be at least partially explained by cancer-type specific effects. DRG-1 expression as well as the expression of other tumor antigens may be differentially regulated by tumor-specific oncogenes or tumor suppressor genes, tumor vascularity, tumor hypoxia, etc. Thus, whether DRG-1 plays a beneficial or detrimental role may be dependent on the tumor microenvironment.

The molecular mechanisms underlying the oncogenic role of DRG-1 remain unclear. Several studies have indicated that DRG-1 may influence cancer growth and progression by modulating angiogenesis. Furthermore, DRG-1 expression is correlated with microvessel density, a measure of tumor angiogenesis, in patients with cervical adenocarcinoma and pancreatic cancer [45,51]. DRG-1 expression also influenced the production of potent angiogenic factors, including IL-8 and vascular endothelial growth factor [43,51]. Recent evidence suggests that DRG-1 targets key signaling pathways in oncogenesis including TGF-β, phosphoinositide 3-kinase, and Ras pathways [53]. The molecular mechanisms of DRG-1 in cancer development and progression require further investigation.

In conclusion, we identified DRG-1 as a melanoma-associated antigen recognized by CD4^+^ Th1 cells. DRG-1 may represent a potentially useful target for the development of immunotherapy regimens for melanoma patients.
